# LDAR shows more consistent prognostic performance than other albumin-derived indices for 28-day ICU mortality in critically ill patients with urosepsis: evidence from a dual-cohort retrospective study

**DOI:** 10.3389/fmed.2026.1915095

**Published:** 2026-10-01

**Authors:** Yi Mu, Hao Tang, Yan Zeng, Qian Wang, Tao Huang, Wei Li, Xiangbo Wu, Shengming Pi

**Affiliations:** 1 Department of Urology, Guiyang Public Health Clinical Center, Guiyang, China; 2 Department of Urology, the Affiliated Hospital of Guizhou Medical University, Guiyang, China

**Keywords:** albumin-derived indices, critical care, deep learning, nutritional intervention, risk stratification

## Abstract

**Background:**

Urosepsis remains a leading cause of ICU mortality. Albumin-derived composite indices, which integrate nutritional, inflammatory, and metabolic parameters, hold prognostic promise; however, their comparative performance has not been directly assessed.

**Methods:**

We conducted a dual-cohort retrospective study using MIMIC-IV (*n* = 3,060) for derivation and the Affiliated Hospital of Guizhou Medical University (*n* = 353) for validation. The primary outcome was 28-day ICU mortality in a competing-risks framework with discharge alive as competing event. Six indices—lactate dehydrogenase-to-albumin ratio (LDAR), anion gap-to-albumin ratio (AGAR), glucose-to-albumin ratio (GAR), red cell distribution width-to-albumin ratio (RAR), total bilirubin-to-albumin ratio (TAR), and urea nitrogen-to-albumin ratio (UAR)—were compared via cause-specific Cox and Fine-Gray subdistribution hazard models, cumulative incidence functions, restricted cubic splines (RCS), Kaplan-Meier curves, and ROC/Youden/cNRI analyses. LASSO-selected features trained a multilayer perceptron (MLP), benchmarked against six severity scores; a parsimonious external model (events per variable ≥ 10) was also examined.

**Results:**

In the derivation cohort, logLDAR, RAR, TAR, AGAR, and UAR were independently associated with 28-day ICU mortality in fully adjusted models (HRs = 1.292, 1.147, 1.136, 1.074, and 1.013, respectively; all *P* < 0.01), whereas logGAR was not. Fine-Gray models corroborated these findings (logLDAR sHR = 1.351, 95% CI: 1.238–1.473). RCS revealed a non-linear logLDAR–mortality relationship (*P* non-linear = 0.040), with risk accelerating beyond logLDAR > 7. logLDAR yielded the highest AUC (0.638, 95% CI: 0.609–0.667) and cNRI (0.119). External validation confirmed the independent prognostic value of logLDAR (HR = 1.524, 95% CI: 1.225–1.895; Fine-Gray sHR = 1.545, 95% CI: 1.295–1.844), with an external AUC of 0.735 (95% CI: 0.648–0.808), although between-cohort differences in case definitions may partly contribute. The MLP with eight LASSO-selected features achieved an external AUC of 0.762 (95% CI: 0.687–0.840), comparable to SOFA plus logLDAR (AUC = 0.782; DeLong *P* = 0.512). SHAP analysis ranked logLDAR among the top predictors.

**Conclusion:**

Among the six albumin-derived indices, logLDAR demonstrated the most consistent and generalizable prognostic performance for 28-day ICU mortality in urosepsis, reflecting both tissue hypoxia and nutritional-inflammatory depletion, and remained robust under competing-risk adjustment. logLDAR may serve as a simple bedside biomarker for early risk stratification; however, prospective studies are warranted to determine whether interventions targeting its underlying pathways can improve outcomes.

## Introduction

Urosepsis is a complex systemic inflammatory response syndrome triggered by urinary tract infection (UTI). Its incidence is rapidly increasing with the growing number of urological procedures and operations, as well as the rising antimicrobial resistance of pathogens. Consequently, urosepsis has become a leading cause of death in non-cardiac intensive care units (ICUs), imposing a heavy burden on families and healthcare systems (1, 2). Early identification of high-risk patients, timely intervention and accurate prognosis assessment are essential to reduce mortality, alleviate organ damage and manage complications (3, 4). Therefore, discovering reliable biomarkers for early warning of poor outcomes in urosepsis has become a research priority for strengthening preventive measures and optimizing treatment strategies (5).

In recent years, the role of clinical nutritional intervention in the acute management of critically ill patients has received increasing attention. The patient’s metabolic and nutritional status directly affects the intensity of the inflammatory response, immune function and tissue repair capacity. However, most traditional clinical scoring systems do not include nutritional-metabolic parameters (6, 7). Serum albumin (ALB) is the most abundant protein in human circulation. It is a key indicator for assessing nutritional status and also plays central roles in maintaining colloid osmotic pressure, mediating anti-inflammatory and antioxidant processes (8, 9). Existing evidence shows that hypoalbuminemia is associated with poor outcomes in various critical illnesses, including sepsis (10, 11).

Nevertheless, as an independent predictor, serum albumin concentration is easily influenced by confounders such as liver function, fluid balance, acute-phase reactions and clinical interventions (e.g., fluid resuscitation, nutritional support). This may compromise the stability and specificity of its prognostic value (12). To overcome the inherent limitations of a single marker, recent studies have increasingly focused on combining albumin with other pathophysiologically relevant and complementary laboratory parameters. Such composite indices may more comprehensively reflect the patient’s inflammatory burden, nutritional reserve, metabolic stress and degree of organ dysfunction, thereby potentially improving clinical utility (13, 14).

Several albumin-derived composite indices have been proposed and applied to prognosis assessment in critical illness. These indices combine albumin with markers from different pathophysiological pathways, thus reflecting different dimensions of the nutrition-inflammation-metabolism-organ function axis. The six indices of interest in the present study are: lactate dehydrogenase/albumin ratio (LDAR), which integrates information on tissue hypoxia and cellular injury (15, 16); anion gap/albumin ratio (AGAR), reflecting metabolic acidosis and internal environment disturbance (17); glucose/albumin ratio (GAR), representing metabolic stress and protein nutritional status (18); red cell distribution width/albumin ratio (RAR), linking systemic inflammation, erythropoiesis disorders and nutritional depletion (19, 20); total bilirubin/albumin ratio (TAR), indicating liver function reserve and jaundice burden (21); and urea nitrogen/albumin ratio (UAR), reflecting kidney function status and protein catabolism (22). These six indicators target different key pathological links during the progression of urosepsis: tissue hypoxia (LDAR), acid-base imbalance (AGAR), metabolic stress (GAR), inflammation and hematopoietic disturbance (RAR), liver dysfunction (TAR), and protein hypercatabolism (UAR). However, no systematic evaluation or head-to-head comparison of these six indices has yet been performed in this specific patient population. Their differential predictive abilities, relative advantages and optimal clinical choices remain largely uncertain (23, 24).

Given that prospective cohort studies require substantial resources, using retrospective data to determine which composite index combines ease of use with stable predictive power is a necessary prerequisite. Such an approach can inform the design of subsequent prospective studies and optimize resource allocation. Therefore, this study aimed to systematically evaluate and compare the independent prognostic value of LDAR, AGAR, GAR, RAR, TAR and UAR for 28-day ICU mortality in patients with urosepsis, using a public database and a real-world cohort for external validation. Through this comprehensive comparison, we sought to identify the index that best combines simplicity and stable prognostic performance for early risk stratification. This would help clinicians identify high-risk patients early, providing an objective basis for prognostic assessment. Whether interventions targeting the pathophysiological pathways captured by these indices can improve outcomes remains to be tested in prospective trials.

## Materials and methods

### Data sources

This study was based on two retrospective cohorts. The internal derivation cohort came from the MIMIC-IV database, a publicly accessible critical care database from the Massachusetts Institute of Technology (25). It contains information on 196,527 adult patients admitted to the Beth Israel Deaconess Medical Center between 2008 and 2019. Access to the data was granted after application approval. Because the analysis involved only de-identified information, institutional review board (IRB) approval was not required. The external validation cohort comprised patients with urosepsis hospitalized in the Department of Urology and the Department of Critical Care Medicine at the Affiliated Hospital of Guizhou Medical University between January 2020 and January 2025. The construction of this cohort was approved by the hospital ethics committee.

### Study population

We focused on patients admitted to the ICU for sepsis caused by UTI. Sepsis was defined according to the Sepsis-3 consensus criteria (26), which were applied retrospectively to all patients in the MIMIC-IV cohort (admissions 2008-2019) using the recorded SOFA scores, lactate values and vasopressor data; although Sepsis-3 was published in 2016, the requisite variables were available throughout the entire study period, allowing uniform case identification. The diagnostic criteria for UTI differed between the two cohorts. In the internal cohort, urosepsis cases were identified by searching for urinary tract infection ICD-9 codes (599.0, 590.10, 590.80, 595.0, 595.9) and ICD-10 codes (N39.0, N10, N11.0, N12, N30.0, N30.9). In the external cohort, urosepsis was diagnosed based on clinical presentation, a positive urine culture ( ≥ 105 CFU/mL), the Sepsis-3 criteria and the absence of any other infection source; all cases were reviewed by two senior urologists. In both cohorts, patients with infections at other sites were excluded to ensure that the urinary tract was the sole source of infection. Patients were included if they met all of the following criteria: age ≥ 18 years, ICU stay ≥ 24 h, and available baseline measurements of all component variables of the six albumin-derived indices, namely LDH, anion gap, glucose, RDW, total bilirubin, urea nitrogen and albumin.

### Data extraction and handling of missing data

After obtaining the necessary permissions, data were extracted using PostgreSQL (13.7.2), Navicat Premium (16.0) and Structured Query Language (SQL). The extracted variables were divided into six categories: demographic characteristics, comorbidities, vital signs, laboratory parameters, illness severity scores and therapeutic measures. For missing data, variables with a missing rate > 30% were excluded. For the remaining variables with missing values, multiple imputation was performed using the “mice” package (version 3.16.0) in R (27). The imputation model included all variables needed for the primary analysis: continuous variables requiring imputation, the fully observed exposure variables (the albumin-derived composite indices and their components), the fully observed outcome variable (28-day mortality), and other fully observed categorical and continuous covariates. This approach maximized the use of available information to predict missing values and strengthened the plausibility of the missing-at-random (MAR) assumption.

### Exposure and clinical outcome

Based on previous literature, we selected six albumin-derived composite indices to comprehensively evaluate inflammatory activation, nutritional status and metabolic stress: LDAR, AGAR, GAR, RAR, TAR and UAR. To satisfy statistical assumptions and improve clinical interpretability, LDAR was log2-transformed and expressed as logLDAR throughout the analysis. The primary endpoint was 28-day ICU mortality, defined as death within 28 days after ICU admission. Time zero was the time of ICU admission. The target estimand was the absolute risk (cumulative incidence) of ICU death by day 28. Because discharge alive from the ICU is associated with a better prognosis and competes with the risk of death, we treated ICU discharge alive as a competing event. Patients who were discharged alive from the ICU before day 28 were considered to have experienced the competing event at the time of ICU discharge; those still in the ICU on day 28 were censored at day 28 if they were alive. Both cause-specific Cox models (for the etiologic, cause-specific hazard question) and Fine-Gray subdistribution hazard models (for the prognostic, absolute-risk question) were fitted, and cumulative incidence functions were estimated.

### Association between albumin-derived indices and 28-day ICU mortality

To control for potential confounders, three sequential adjustment models were constructed. Model 1 had no adjustment. Model 2 was adjusted for demographic variables (age, sex, race, weight). Based on previous literature (28), Model 3 was further adjusted for a pre-specified set of variables, including major comorbidities [acute kidney injury (AKI), chronic kidney disease (CKD), diabetes mellitus (DM), ischaemic heart disease (IHD), chronic obstructive pulmonary disease (COPD)], vital signs [heart rate (HR), non-invasive blood pressure systolic (NBPS), non-invasive blood pressure diastolic (NBPD), respiratory rate (RR)], laboratory parameters [hematocrit (HCT), platelets (PLT), white blood cells (WBC), calcium (Ca), glucose (Glu), potassium (K), total carbon dioxide (TCO2), pH, prothrombin time (PT), partial thromboplastin time (PTT), alanine aminotransferase (ALT)], and treatments [serum albumin administration (SA), vasopressors (VP), continuous renal replacement therapy (CRRT), ventilation, glucocorticoids GC)]. In addition, Model 3 included the component molecules of the other albumin-derived indices [red cell distribution width (RDW), total bilirubin (TB), creatinine (CREA), anion gap (GAP), urea nitrogen (UREA), lactate dehydrogenase (LDH)] to fully account for confounding. Importantly, to avoid multicollinearity with the index being studied, the component variables of each specific albumin-derived index (e.g., LDH and albumin for LDAR; RDW and albumin for RAR; total bilirubin and albumin for TAR, etc.) were intentionally excluded from Model 3 when that index served as the exposure. The variance inflation factor (VIF) was calculated for all remaining covariates, and no VIF exceeded 5, indicating no significant multicollinearity. Furthermore, restricted cubic spline (RCS) regression (29) was used, with knots placed at the 5th, 35th, 65th and 95th percentiles, to explore non-linear relationships between the albumin-derived indices and the endpoint.

### Comparison of albumin-derived indices

The albumin-derived composite indices were compared across four dimensions: (1) AUC with bootstrap 95% confidence intervals (CIs) and pairwise DeLong tests (30) across 15 comparisons; (2) the optimal cut-off determined by the Youden index (J = max[sensitivity + specificity – 1]) in the derivation cohort, and diagnostic performance evaluated at that threshold in both the derivation and external validation cohorts; (3) adding each index to each of the six severity scores (36 combinations) and using the mean cNRI (30, 31) across the scores as a summary measure; and (4) the aforementioned association analysis between each albumin-derived index and 28-day ICU mortality.

### Deep learning prediction model

Feature selection was performed using LASSO (L1 regularization) (32) regression with 10-fold cross-validation on 34 candidate features: 27 laboratory variables that showed significant univariate association with ICU mortality (Mann-Whitney U test, BH-FDR < 0.05) and 7 albumin-derived indices. The optimal regularization parameter (α = 0.016) was determined using the 1-SE rule, i.e., selecting the simplest model within one standard error of the minimum cross-validated mean squared error. Features with non-zero coefficients at that α were retained. No *a priori* exclusion was applied to index component variables; the L1 penalty automatically handled collinearity by shrinking redundant coefficients to zero. A multilayer perceptron (MLP) (33) with three hidden layers (128, 64 and 32 neurons), ReLU activation and dropout (rate = 0.5) was trained using weighted binary cross-entropy loss (pos_weight = 5.22) to address class imbalance (event rate 16.1%). Optimization used Adam (lr = 0.001, weight_decay = 1e-4) with ReduceLROnPlateau scheduling and early stopping (patience = 20 epochs). The derivation cohort was randomly split (seed = 42) into a training set (60%, *n* = 1,836) and a validation set (40%, *n* = 1,224). Features were standardized using z-score normalization fitted on the training set. The model was evaluated on the training set, validation set, full derivation cohort and independent external cohort (*n* = 353). Performance metrics included AUC, sensitivity, specificity, positive predictive value (PPV), negative predictive value (NPV), F1 score, Youden index and Brier score. Feature importance was assessed using SHapley Additive exPlanations (SHAP) (34) with KernelExplainer (100 background samples, 300 evaluation samples, n_samples = 100, seed = 42).

### Statistical analysis

Continuous variables were summarized as mean ± SD or median (IQR) based on normality, with group comparisons by t-test, ANOVA, or Kruskal-Wallis; categorical variables as *n* (%) and compared by chi-squared or Fisher’s exact test. All analyses used R 4.5.1, with two-sided *P* < 0.05 significant. For competing risks (ICU discharge alive as competing event), Fine-Gray subdistribution models (‘cmprsk’ package) were fitted; cumulative incidence functions were estimated and compared by Gray’s test across logLDAR median strata. Subdistribution and cause-specific HRs are reported. For external validation (40 events, *n* = 353), the full model (34 covariates) yielded EPV≈1.2, risking biased estimates. Therefore, a parsimonious model was pre-specified with age, sex, AKI (largest baseline difference), and logLDAR (EPV = 10). LASSO-penalized Cox with 10-fold CV and 100 bootstrap resamples assessed feature stability. For the MLP, bootstrap 95% CIs for AUC and calibration were reported; a logistic model combining SOFA and logLDAR was fitted for direct comparison.

## Results

### Baseline characteristics of the study population

Table 1 summarizes baseline characteristics by ICU mortality. In the derivation cohort (*n* = 3,060, 15.4% died), non-survivors were older (median 76.0 vs. 72.0 years, *P* < 0.001), had a lower proportion of white race (*P* = 0.022), and had higher rates of AKI (77.3% vs. 60.0%) and MI (14.2% vs. 9.6%) but a lower rate of hyperlipidaemia (27.6% vs. 36.5%) (all *P* < 0.05). All severity scores (SOFA, APS III, SAPS II, OASIS, Charlson, APACHE II) were higher in non-survivors (all *P* < 0.001). They also had lower systolic/diastolic blood pressures and oxygen saturation (*P* ≤ 0.01–0.021), while heart rate and respiratory rate did not differ. Laboratory findings: non-survivors showed higher RDW, WBC, anion gap, potassium, lactate, INR, PT, PTT, ALT, AST, bilirubin, creatinine, urea and LDH, and lower albumin, hemoglobin, red blood cells, platelets, hematocrit, total CO2 and pH (all *P* < 0.05); calcium, chloride, glucose and sodium were similar. They more often received vasoactive drugs (78.8% vs. 59.4%), vasopressors (77.9% vs. 65.1%), glucocorticoids (39.5% vs. 31.2%) and CRRT (18.3% vs. 9.1%) (all *P* < 0.001). Among albumin-derived indices, LDAR, log(LDAR), RAR, AGAR, UAR and TAR were higher in non-survivors (all *P* < 0.001), whereas GAR and log(GAR) showed no difference (*P* > 0.05). In the external validation cohort (*n* = 353, 11.3% died), LDAR, log(LDAR), AGAR, UAR and TAR were significantly higher in non-survivors (all *P* < 0.05), but RAR, GAR and log(GAR) were not.

**TABLE 1:** Baseline characteristics stratified by ICU mortality.

Variable	Survived (*n* = 2,589)	Died (*n* = 471)	*P*
Age	72.00 [61.00–82.00]	76.00 [63.00–84.50]	< 0.001
Weight	77.90 [64.50–95.40]	76.60 [63.15–95.70]	0.389
Gender	1129 (43.6%)	200 (42.5%)	0.645
AKI	1554 (60.0%)	364 (77.3%)	< 0.001
CKD	739 (28.5%)	150 (31.8%)	0.146
DM	995 (38.4%)	160 (34.0%)	0.066
HLD	944 (36.5%)	130 (27.6%)	< 0.001
HF	1001 (38.7%)	200 (42.5%)	0.120
MI	249 (9.6%)	67 (14.2%)	0.003
IHD	948 (36.6%)	178 (37.8%)	0.627
COPD	406 (15.7%)	86 (18.3%)	0.161
Hpy	934 (36.1%)	143 (30.4%)	0.017
Race	1698 (65.6%)	283 (60.1%)	0.022
SOFA	6.00 [4.00–9.00]	8.00 [6.00–11.00]	< 0.001
APSIII	55.00 [43.00–68.00]	68.00 [52.00–84.00]	< 0.001
SAPSII	43.00 [35.00–52.00]	52.00 [41.50–61.00]	< 0.001
OASIS	35.00 [30.00–41.00]	38.00 [33.00–44.00]	< 0.001
Charlson	6.00 [4.00–8.00]	7.00 [5.00–9.00]	< 0.001
APACHEII	21.00 [17.00–26.00]	24.00 [19.00–29.00]	< 0.001
HR	90.00 [76.00–105.00]	91.00 [78.00–105.00]	0.308
NBPS	119.00 [102.00–138.00]	113.00 [99.00–132.00]	< 0.001
NBPD	65.00 [55.00–78.00]	62.00 [52.00–77.00]	0.010
RR	19.00 [16.00–23.00]	20.00 [16.00–24.00]	0.437
Spo2	98.00 [95.00–100.00]	97.00 [94.00–100.00]	0.021
HCT	31.10 [26.80–35.60]	30.30 [25.45–35.20]	0.014
Hb	10.00 [8.60–11.60]	9.80 [8.20–11.40]	0.015
PLT	184.00 [124.00–258.00]	165.00 [105.00–247.00]	0.005
RDW	15.50 [14.10–17.20]	16.30 [14.60–18.60]	< 0.001
RBC	3.35 [2.89–3.92]	3.27 [2.73–3.83]	0.002
WBC	11.60 [7.90–16.10]	13.00 [8.40–18.70]	< 0.001
ALB	2.90 [2.50–3.30]	2.70 [2.30–3.20]	< 0.001
GAP	15.00 [12.00–18.00]	16.00 [13.00–19.00]	0.005
Ca	8.30 [7.70–8.80]	8.20 [7.60–8.80]	0.152
Cl	104.00 [99.00–109.00]	104.00 [98.00–109.00]	0.341
Glu	133.00 [106.00–175.00]	134.00 [100.00–175.50]	0.180
K	4.10 [3.70–4.60]	4.20 [3.70–4.80]	0.007
Na	139.00 [135.00–142.00]	139.00 [134.00–142.00]	0.409
TCO2	24.00 [20.00–28.00]	23.00 [18.00–27.00]	< 0.001
Lac	1.70 [1.20–2.70]	2.10 [1.50–3.30]	< 0.001
PH	7.36 [7.29–7.42]	7.35 [7.26–7.41]	< 0.001
PO2	84.00 [48.00–151.00]	77.00 [47.00–124.00]	0.028
INR	1.30 [1.20–1.70]	1.50 [1.20–2.00]	< 0.001
PT	14.80 [12.90–18.50]	16.50 [13.75–22.00]	< 0.001
PTT	32.10 [27.60–40.30]	35.00 [28.90–46.95]	< 0.001
ALT	25.00 [15.00–52.00]	28.00 [17.00–62.50]	0.002
AST	38.00 [22.00–81.00]	51.00 [27.00–114.50]	< 0.001
TB	0.60 [0.40–1.30]	0.90 [0.40–2.70]	< 0.001
CREA	1.30 [0.80–2.20]	1.50 [0.90–2.40]	0.006
UREA	28.00 [17.00–48.00]	36.00 [22.00–57.00]	< 0.001
LDH	278.00 [210.00–396.00]	348.00 [247.00–546.50]	< 0.001
LDAR	96.67 [70.73–144.23]	129.66 [87.42–219.85]	< 0.001
logLDAR	6.59 [6.14–7.17]	7.02 [6.45–7.78]	< 0.001
GAR	45.94 [35.59–64.19]	49.60 [36.53–68.15]	0.053
logGAR	3.83 [3.57–4.16]	3.90 [3.60–4.22]	0.052
RAR	5.40 [4.48–6.57]	6.08 [5.09–7.71]	< 0.001
AGAR	5.17 [4.17–6.50]	5.71 [4.71–7.31]	< 0.001
UAR	9.67 [5.71–17.35]	13.02 [7.78–22.95]	< 0.001
TAR	0.22 [0.12–0.48]	0.31 [0.16–1.07]	< 0.001
SA	1685 (65.1%)	367 (77.9%)	< 0.001
VP	1537 (59.4%)	371 (78.8%)	< 0.001
GC	809 (31.2%)	186 (39.5%)	< 0.001
Ventilation	2228 (86.1%)	409 (86.8%)	0.652
CRRT	235 (9.1%)	86 (18.3%)	< 0.001

Demographic, clinical, laboratory, and treatment characteristics of the derivation cohort (MIMIC-IV, *n* = 3,060) and external validation cohort (Guizhou Medical University, *n* = 353), stratified by ICU mortality status. Continuous variables are presented as median [interquartile range] or mean ± standard deviation; categorical variables as *n* (%). *P*-values from Mann-Whitney U test, Student *t*-test, or chi-square test as appropriate.

### Association between albumin-derived indices and 28-day ICU mortality

Cox regression showed that in the derivation cohort (Table 2), five indices—logLDAR, RAR, TAR, AGAR, and UAR—were independently associated with 28-day ICU mortality in fully adjusted Model 3 (HRs 1.292, 1.147, 1.136, 1.074, 1.013, all *P* < 0.01), while logGAR was not (*P* = 0.687). logLDAR exhibited the strongest association. In the external validation, only logLDAR remained significant in the full Model 3 (HR = 2.213, 95% CI: 1.459–3.356), whereas the other five lost significance. However, because the full model had low EPV (∼1.2) and risked overfitting, a parsimonious model (age, sex, AKI, logLDAR; EPV = 10) was pre-specified and confirmed logLDAR’s independent value (HR = 1.524, 95% CI: 1.225–1.895, *P* < 0.001). Competing-risks analysis with Fine-Gray models yielded sHRs consistent with cause-specific HRs: derivation sHR = 1.351 (95% CI: 1.238–1.473) and external parsimonious sHR = 1.545 (95% CI: 1.295–1.844), both *P* < 0.001. Cumulative incidence curves stratified by median logLDAR showed higher mortality in the high-logLDAR group (Gray’s test *P* < 0.001 in both cohorts) (Supplementary Figure 3). These results confirm logLDAR as the most robust prognostic marker among the six indices, even under competing-risk adjustment, and underscore the importance of parsimonious modelling in small validation samples.

**TABLE 2:** Cox proportional hazards regression for ICU mortality (fully adjusted model 3).

Index	Internal HR (95% CI)	*P*	External HR (95% CI)	*P*
logLDAR	1.292 (1.177–1.418)	< 0.001	2.379 (1.422–3.980)	< 0.001
logGAR	0.958 (0.776–1.182)	0.687	0.597 (0.279–1.278)	0.184
RAR	1.147 (1.096–1.201)	< 0.001	0.998 (0.774–1.287)	0.988
AGAR	1.074 (1.024–1.126)	0.003	1.050 (0.839–1.313)	0.669
UAR	1.013 (1.006–1.021)	< 0.001	1.001 (0.971–1.032)	0.951
TAR	1.136 (1.093–1.180)	< 0.001	0.991 (0.794–1.236)	0.935

Hazard ratios (HR) and 95% confidence intervals for the association between albumin-derived indices and 28-day ICU mortality in the fully adjusted Model 3. Model 3 adjusted for demographics (age, sex, race, weight), comorbidities (AKI, CKD, DM, IHD, COPD), vital signs (HR, NBPS, NBPD, RR), laboratory parameters (HCT, PLT, WBC, Ca, Glu, K, TCO2, PH, PT, PTT, ALT), treatments (SA, VP, CRRT, ventilation, GC), and component molecules of other indices (RDW, TB, CREA, GAP, UREA, LDH). Component variables of the index being analyzed were excluded to avoid multicollinearity.

### Dose-response relationship between albumin-derived indices and 28-day ICU mortality

Kaplan–Meier analysis (derivation) showed that (Figure 1), except for logGAR (*P* = 0.147), all five indices significantly stratified ICU survival when dichotomized by their respective medians (logLDAR [median 6.66]: log-rank *P* = 1.07 × 10^−6^; RAR: *P* = 6.20 × 10^−12^; UAR: *P* = 3.59 × 10^−16^; AGAR: *P* = 1.38 × 10^−7^; TAR: *P* = 3.84 × 10^−7^; all *P* < 0.001). In external validation, the stratification remained significant for logLDAR (*P* = 4.46 × 10^−5^), UAR (*P* = 1.30 × 10^−5^), and AGAR (*P* = 9.80 × 10^−4^), but not for TAR (*P* = 0.067), RAR (*P* = 0.114), or logGAR (Figure 2 and Supplementary Figure 1).

**FIGURE 1:**
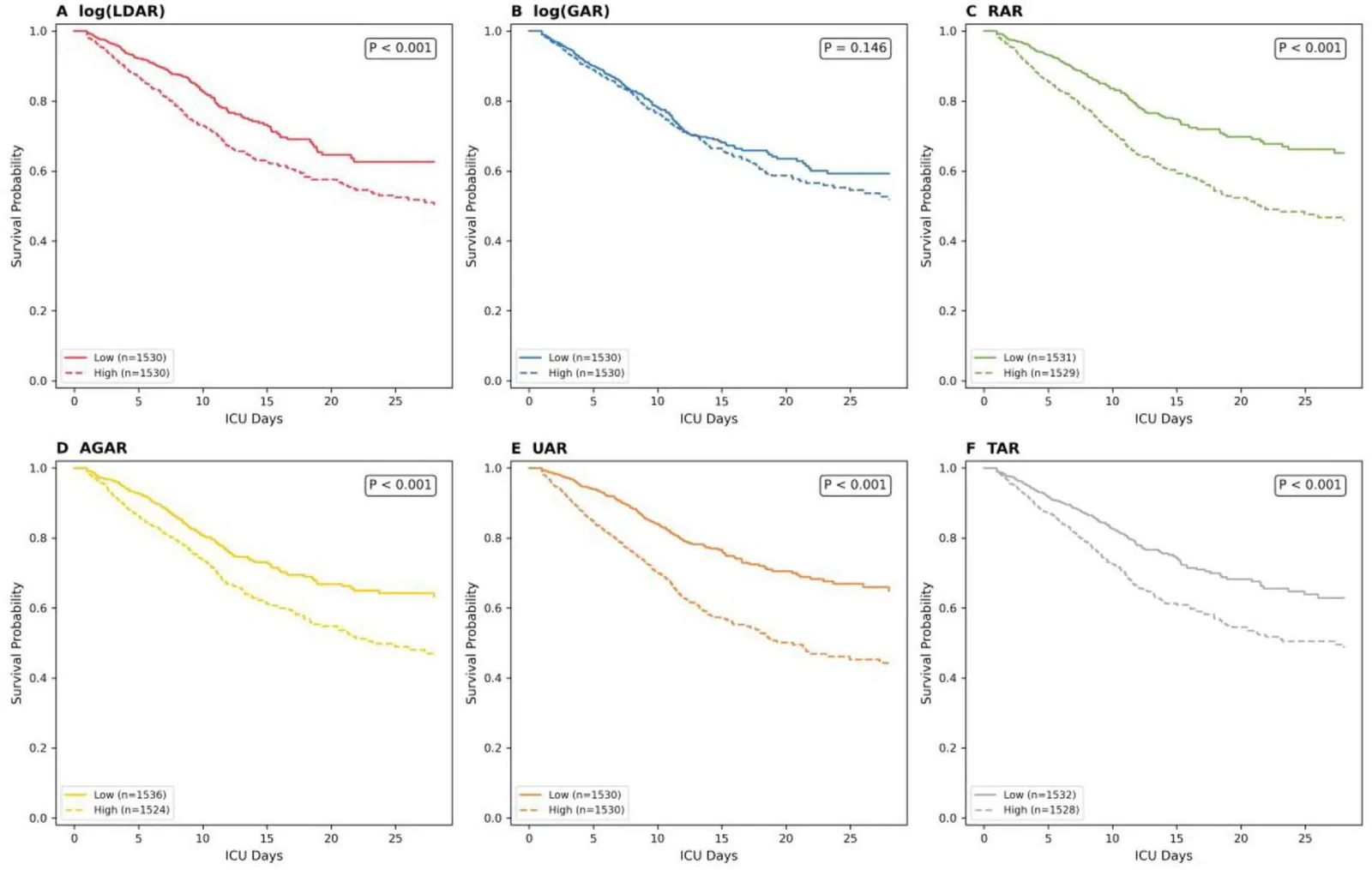
Kaplan-Meier survival curves by albumin-derived indices (derivation cohort). Kaplan-Meier survival curves stratified by median values of six albumin-derived indices in the derivation cohort (*n* = 3,060). **(A)** log(LDAR), log-rank *P* = 1.07 × 10^−6^; **(B)** log(GAR), *P* = 0.147; **(C)** RAR, *P* = 6.20 × 10^−12^; **(D)** AGAR, *P* = 1.38 × 10^−7^; **(E)** UAR, *P* = 3.59 × 10^−16^; **(F)** TAR, *P* = 3.84 × 10^−7^. The median values in the derivation cohort were: logLDAR = 6.66 (LDAR = 100.91), logGAR = 5.53 (GAR = 46.32), RAR = 5.47, AGAR = 5.22, UAR = 10.35, TAR = 0.23. Red lines = above-median group; Blue lines = below-median group. Shaded areas represent 95% confidence intervals.

**FIGURE 2:**
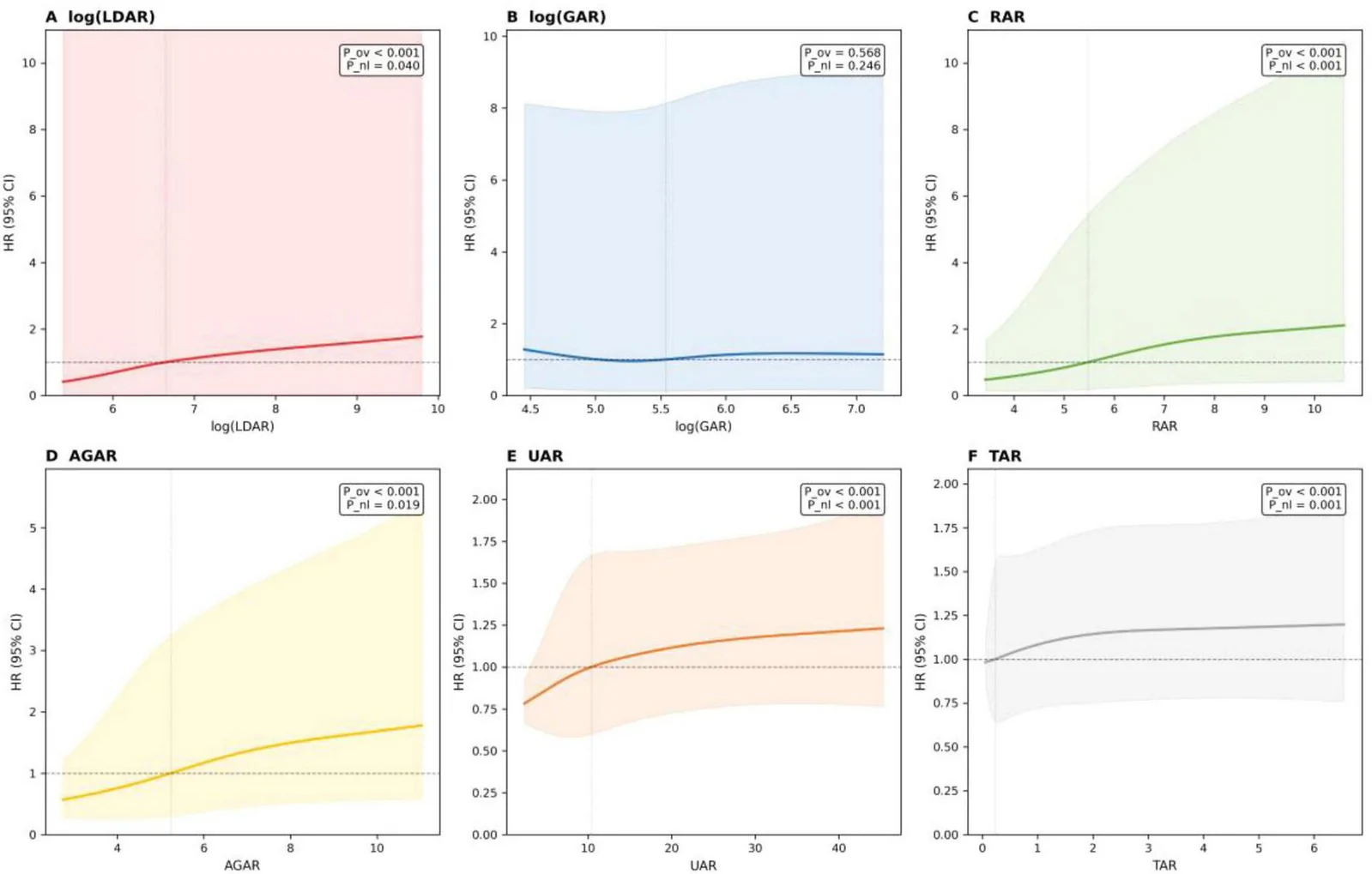
Restricted cubic spline analysis of albumin-derived indices and ICU mortality (derivation cohort). Restricted cubic spline (RCS) curves showing the dose-response relationships between six albumin-derived indices and 28-day ICU mortality in the derivation cohort (*n* = 3,060). **(A)** log(LDAR), *P* overall < 0.001, *P* nonlinear = 0.040; **(B)** log(GAR), *P* overall = 0.568, *P* nonlinear = 0.246; **(C)** RAR, *P* nonlinear < 0.001; **(D)** AGAR, *P* nonlinear = 0.019; **(E)** UAR, P nonlinear < 0.001; **(F)** TAR, *P* nonlinear = 0.001. Knots placed at the 5th, 35th, 65th, and 95th percentiles. Shaded areas represent 95% confidence intervals. HR = hazard ratio; dashed line = null reference (HR = 1).

Restricted cubic spline analyses in the derivation cohort revealed significant non-linear relationships for logLDAR (overall *P* < 0.001, nonlinear *P* = 0.040), RAR, UAR, TAR, and AGAR (all nonlinear *P* < 0.05), with logLDAR exhibiting a threshold effect—risk accelerated sharply beyond logLDAR > 7. In contrast, logGAR showed neither overall (*P* = 0.568) nor nonlinear (*P* = 0.246) significance. External validation confirmed the non-linear patterns for logLDAR (overall *P* < 0.001, nonlinear *P* = 0.037), AGAR (overall *P* < 0.001, nonlinear *P* = 0.036), and UAR (overall *P* < 0.001, nonlinear *P* = 0.012), whereas RAR, TAR, and logGAR did not reach significance (Supplementary Figure 2).

### Comparison of predictive performance and incremental value of albumin-derived indices

ROC analysis (Figures 3A,B) showed that logLDAR had the highest AUC for predicting ICU death in the derivation cohort (0.638, 95% CI: 0.609–0.667), followed by RAR (0.623), UAR (0.607), TAR (0.600), AGAR (0.591) and logGAR (0.528). DeLong tests indicated that logLDAR significantly outperformed logGAR and AGAR (*P* = 0.001 and 0.008, respectively), but not RAR, UAR or TAR (*P* ≥ 0.064). In external validation, logLDAR’s AUC rose to 0.735 (0.648–0.808), significantly exceeding logGAR (0.523, *P* < 0.001) and RAR (0.576, *P* = 0.023), yet comparable to UAR, AGAR, and TAR. These external estimates should be interpreted cautiously given between-cohort differences in case definition (ICD-based vs culture-confirmed). At the optimal Youden cut-off, logLDAR yielded the highest Youden index (0.207) among all indices, with cut-off 7.010, sensitivity 51.0%, specificity 69.7%, and NPV 88.7%; RAR had the highest sensitivity (77.5%) and NPV (90.8%). External validation further increased logLDAR’s Youden index to 0.363 (sensitivity 62.5%, specificity 73.8%, NPV 93.9%).

**FIGURE 3:**
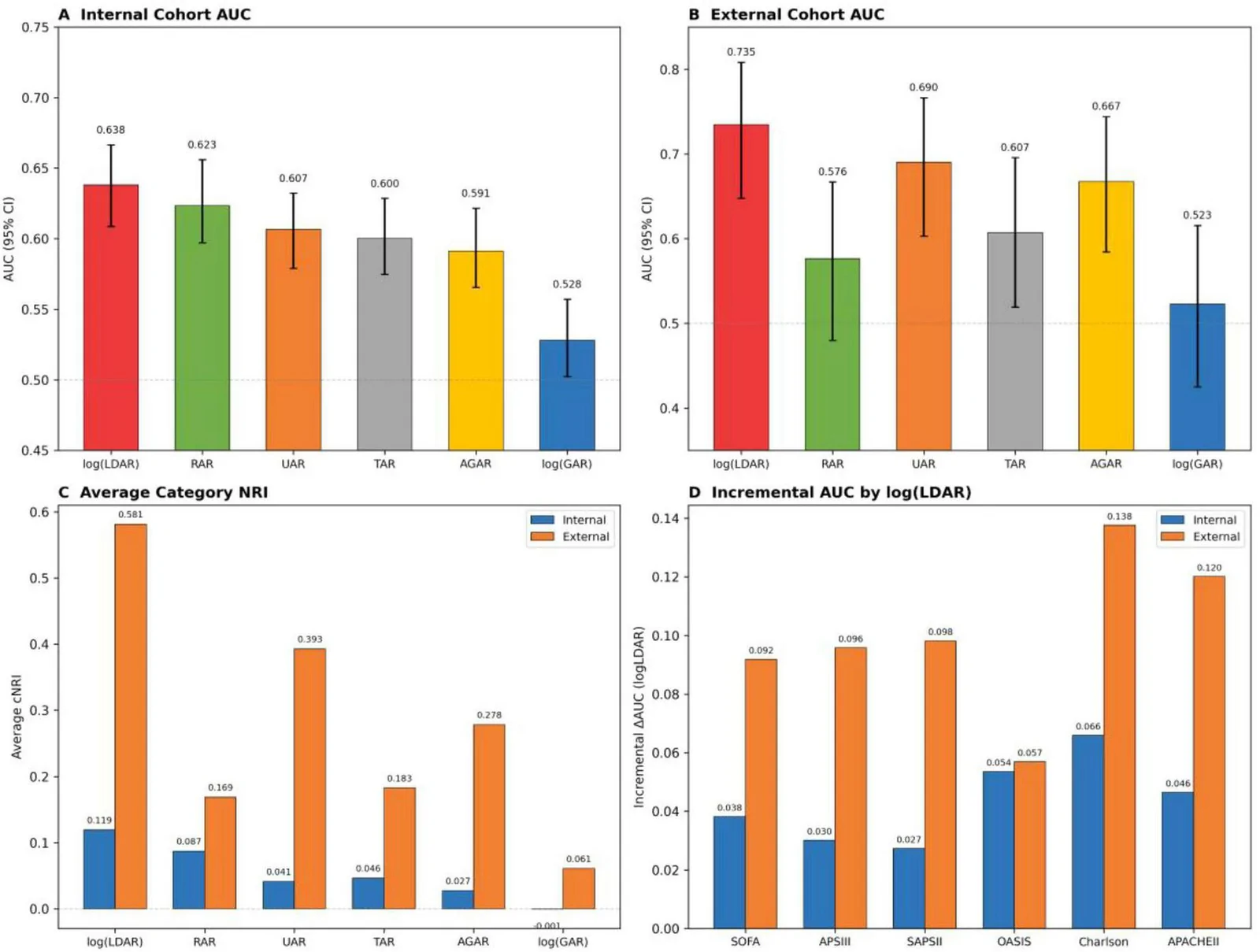
Predictive performance and incremental value of albumin-derived indices. **(A)** ROC curves for six albumin-derived indices in the derivation cohort. logLDAR achieved the highest AUC (0.638). **(B)** ROC curves in the external validation cohort. logLDAR AUC increased to 0.735. **(C)** Incremental AUC (ΔAUC) of each index added to six traditional severity scores in the derivation cohort. **(D)** Incremental AUC in the external validation cohort. logLDAR showed the highest average ΔAUC in both cohorts. Error bars represent 95% confidence intervals from bootstrap resampling.

For incremental value over six severity scores (Figures 3C,D), logLDAR had the highest average cNRI internally (0.119), followed by RAR (0.087), TAR (0.046), UAR (0.041), AGAR (0.027), and logGAR (-0.001); logLDAR was significant for all six scores, whereas logGAR was significant for only one. In external validation, logLDAR’s average cNRI increased to 0.581 (significant for all scores), far above the others (UAR 0.393, AGAR 0.278, TAR 0.183, RAR 0.169, logGAR 0.061). Average incremental AUCs for logLDAR were 0.044 internally and 0.100 externally (all *P* < 0.05). Collectively, logLDAR demonstrated the strongest discriminative ability, independent prognostic value, and reclassification improvement, and was the only index maintaining statistically significant extrinsic validation (Tables 3, 4).

**TABLE 3:** Predictive performance of albumin-derived indices for ICU mortality.

Index	Internal AUC (95% CI)	External AUC (95% CI)	Cutoff	Int Sens/Spec	Ext Sens/Spec	Int Youden
logLDAR	0.638 (0.609–0.667)	0.735 (0.648–0.808)	7.010	0.510/0.697	0.625/0.738	0.207
RAR	0.623 (0.597–0.656)	0.576 (0.480–0.667)	5.036	0.775/0.404	0.725/0.409	0.179
UAR	0.607 (0.579–0.632)	0.690 (0.603–0.766)	10.435	0.635/0.530	0.750/0.553	0.165
TAR	0.600 (0.575–0.629)	0.607 (0.519–0.696)	0.235	0.622/0.531	0.650/0.553	0.153
AGAR	0.591 (0.565–0.622)	0.667 (0.584–0.744)	5.172	0.645/0.496	0.750/0.489	0.142
logGAR	0.528 (0.502–0.557)	0.523 (0.425–0.616)	3.928	0.486/0.593	1.000/0.000	0.079

AUC, optimal cutoff (determined by Youden index in the derivation cohort), sensitivity, specificity, and Youden index for six albumin-derived indices in both the derivation and external validation cohorts. 95% confidence intervals for AUC were calculated using bootstrap resampling. DeLong test was used for pairwise AUC comparisons.

**TABLE 4:** Incremental predictive value of logLDAR over traditional severity scores.

Score	Int AUC	Int AUC+	Int Δ AUC	Ext AUC	Ext AUC+	Ext Δ AUC	Int cNRI	Ext cNRI
SOFA	0.635	0.673	0.038	0.690	0.782	0.092	0.062	0.643
APSIII	0.663	0.693	0.030	0.672	0.767	0.096	0.072	0.517
SAPSII	0.671	0.698	0.027	0.678	0.776	0.098	0.061	0.575
OASIS	0.607	0.661	0.054	0.749	0.806	0.057	0.194	0.449
Charlson	0.593	0.659	0.066	0.617	0.755	0.138	0.198	0.689
APACHEII	0.630	0.676	0.046	0.637	0.757	0.120	0.127	0.613

Incremental AUC (ΔAUC) and category-free net reclassification improvement (cNRI) when logLDAR was added to each of six traditional severity scores. AUC = baseline AUC of the score; AUC+ = AUC after adding logLDAR; ΔAUC = AUC+ - AUC. All ΔAUC values were statistically significant (*P* < 0.001 for internal; *P* ≤ 0.002 for external). cNRI *P* < 0.05 for all scores in both cohort.

### Development and validation of a deep learning prediction model

LASSO with 10-fold cross-validation (1-SE rule, λ = 0.016) selected eight non-zero variables: RAR (0.034), logLDAR (0.033), TB (0.021), UAR (0.015), PTT (0.008), PT (0.002), pH (-0.002), and CREA (-0.001) (Figures 4A,B). A four-layer MLP built on these features achieved AUCs of 0.746 (sensitivity 66.1%, specificity 73.9%, Youden 0.400, Brier 0.207) in training, 0.686 (59.1%, 73.2%, 0.323, 0.218) in internal validation, and 0.762 (95% CI: 0.687–0.840; 80.0%, 75.7%, 0.557, 0.196) in external validation (bootstrap 95% CIs from 1000 resamples). The MLP outperformed most individual severity scores (Figures 4C–E), but a logistic model combining SOFA with logLDAR achieved a comparable external AUC of 0.782 (DeLong *P* = 0.512 vs. MLP), suggesting limited added benefit of the full neural network at this sample size. SHAP analysis ranked logLDAR as a positive contributor to death prediction (Figures 5A,B), and decision curves showed higher net benefit over traditional scores for most thresholds (Figures 5C,D). Calibration was reasonable internally; in the external cohort, the Hosmer–Lemeshow test was non-significant (*P* = 0.124), though the calibration slope (4.134) indicated some overfitting (Figure 5E,F and Supplementary Figure 4). LASSO selection stability was modest—logLDAR appeared in 46% of 100 bootstrap resamples, consistent with known instability of penalized selection in small samples.

**FIGURE 4:**
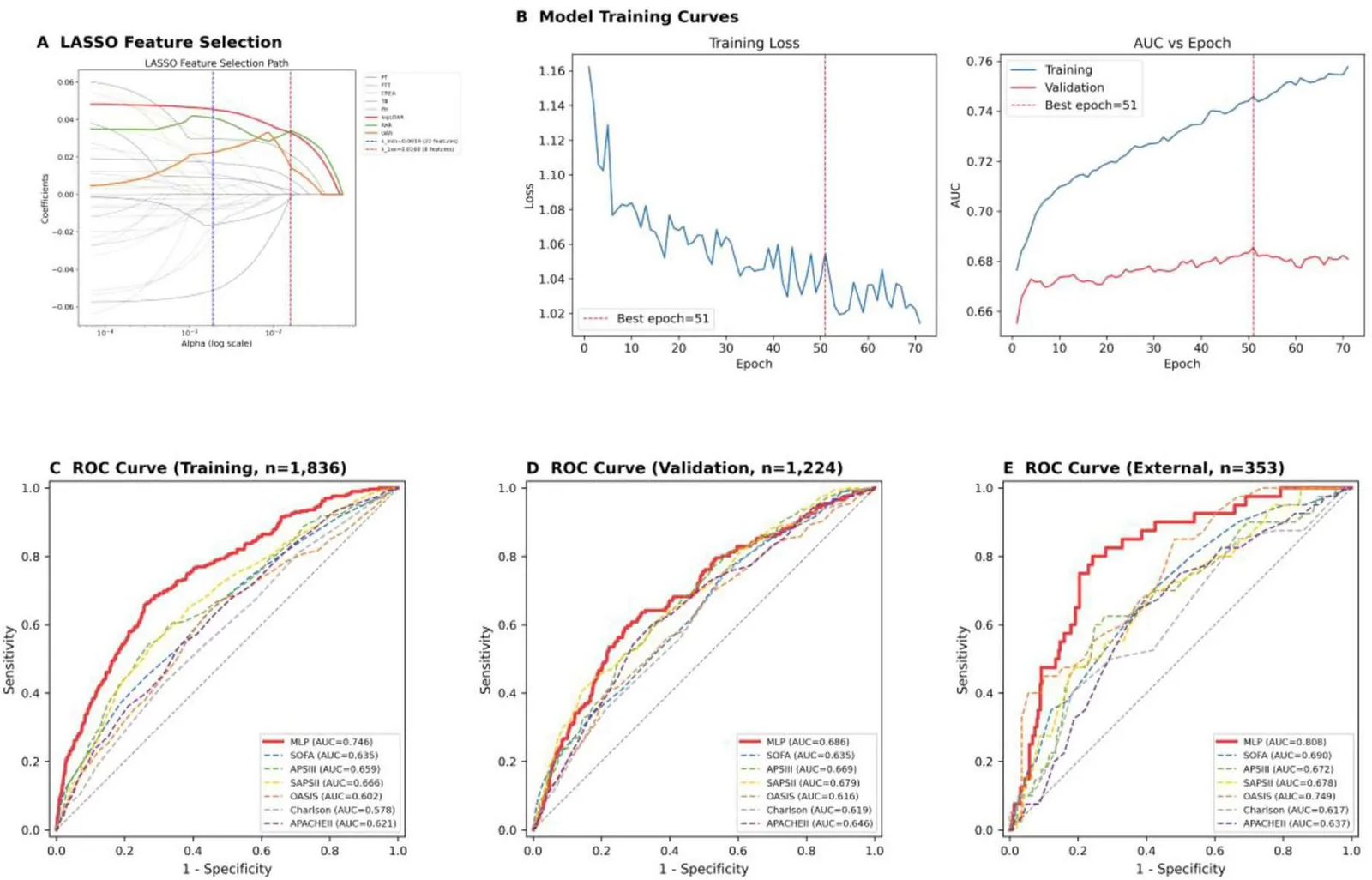
Deep learning model development and validation. **(A)** LASSO regression coefficient paths with 10-fold cross-validation. The vertical dashed line indicates the optimal λ (0.016) selected by the 1-SE criterion, retaining 8 non-zero features. **(B)** Model training curves showing loss and AUC over epochs. Early stopping triggered at epoch 51 (patience = 20). **(C)** ROC curves comparing the MLP model with six traditional severity scores in the training set (*n* = 1,836). MLP AUC = 0.746. **(D)** ROC curves in the validation set (*n* = 1,224). MLP AUC = 0.686. **(E)** ROC curves in the external validation set (*n* = 353). MLP AUC = 0.762 (95% CI: 0.687–0.840). MLP = solid red line; traditional scores = dashed lines. DeLong tests for MLP vs. individual scores are shown in Supplementary Figure 4; the comparison is not like-for-like (multivariable model vs. single score).

**FIGURE 5:**
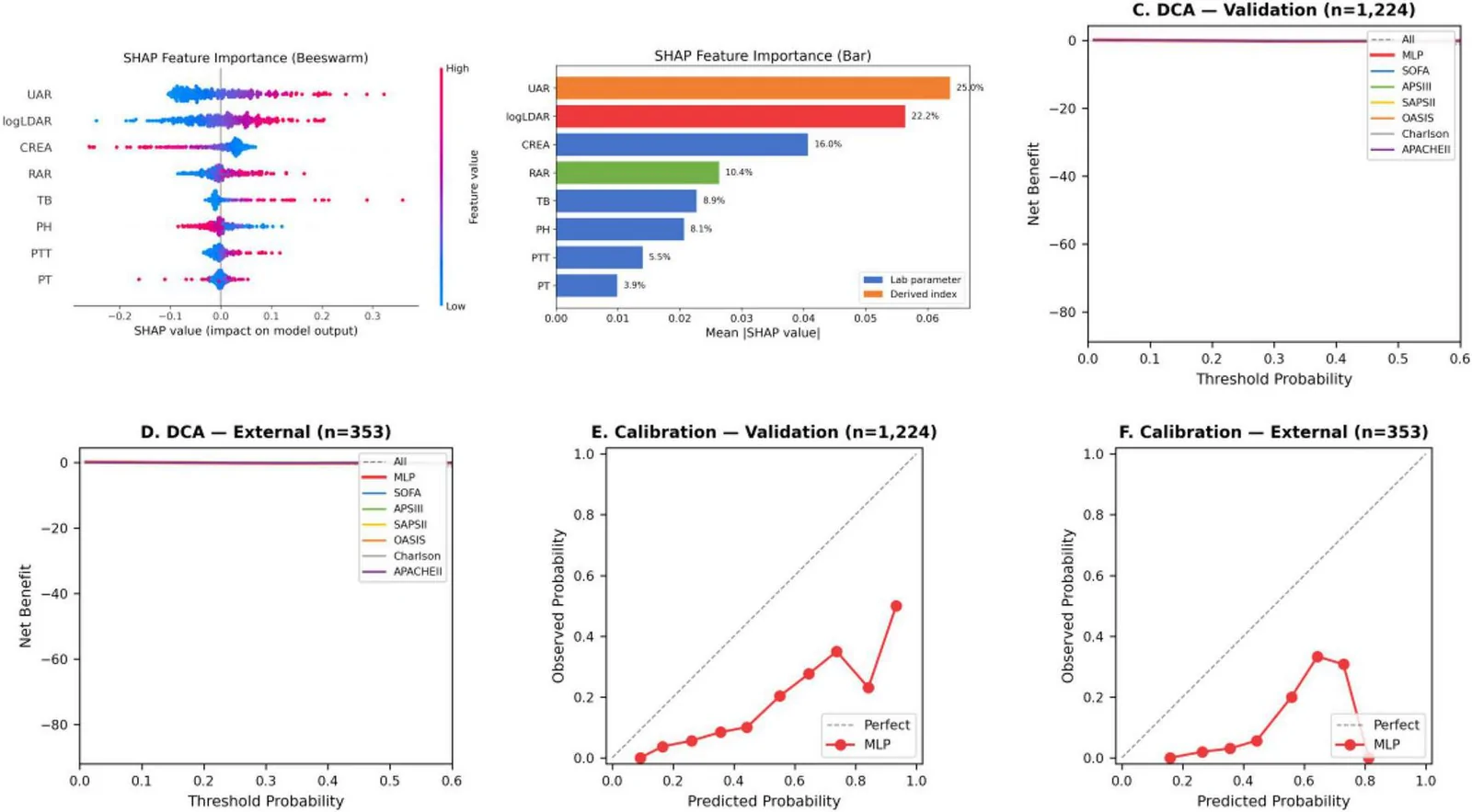
SHAP analysis, decision curve analysis, and calibration curves. **(A)** SHAP beeswarm plot showing feature importance and direction of effect. Each dot represents one patient; color indicates feature value (red = high, blue = low). log (LDAR) shows the strongest positive SHAP values. **(B)** SHAP bar plot of mean absolute SHAP values. log(LDAR) ranks second after RAR in overall contribution. **(C)** Decision curve analysis (DCA) in the validation cohort. The MLP model shows higher net benefit than traditional scores across most threshold probabilities. **(D)** DCA in the external validation cohort. MLP maintains superior net benefit. **(E)** Calibration curve in the validation cohort. Predicted probabilities align closely with observed outcomes. **(F)** Calibration curve in the external validation cohort, confirming good calibration.

## Discussion

This dual-cohort study (derivation *n* = 3,060; external validation *n* = 353) is the first head-to-head comparison of six albumin-derived indices for predicting 28-day ICU mortality in urosepsis. Traditional severity scores such as SOFA and APACHE II are widely used but are cumbersome to calculate. They also do not directly reflect the nutrition-inflammation-metabolism axis that characterizes uroseptic patients (35). Early enteral nutrition guidelines have emphasized the importance of nutritional support in the acute phase of critical illness (36). Our main finding is that logLDAR showed the most consistent prognostic performance across cohorts and statistical models. In the derivation cohort, it was independently associated with mortality in the fully adjusted cause-specific Cox model (HR = 1.292, *P* < 0.001) and in the Fine-Gray subdistribution hazard model (sHR = 1.351, *P* < 0.001). In external validation, the parsimonious model (adjusted for age, sex and AKI; EPV = 10) confirmed this association (HR = 1.524, *P* < 0.001; sHR = 1.545, *P* < 0.001). The other five indices lost independent predictive ability after full adjustment in the external cohort. logLDAR also had the highest AUC (0.638 internally; 0.735 externally) and the highest average cNRI (0.119 internally; 0.581 externally). It showed a clear non-linear threshold at logLDAR > 7, beyond which risk increased sharply.

The advantage of logLDAR may come from its integration of two key pathways in urosepsis progression. LDH is released during cell injury and tissue hypoxia, which are common in endotoxin-induced microcirculatory disturbance (37, 38). Elevated LDH directly reflects the severity of organ damage (39). Albumin, as the denominator, captures nutritional reserve and inflammation-driven capillary leak (40, 41). Therefore, logLDAR simultaneously tracks tissue hypoxia and nutrition-inflammation depletion. Other indices cover only one of these dimensions. For example, RAR reflects chronic inflammation but lags behind acute stress. AGAR and UAR are confounded by acid-base status and renal function, respectively. GAR is confounded by baseline hyperglycaemia in diabetic patients. TAR may have a late predictive window because liver dysfunction typically appears later in urosepsis. Several previous studies validated individual indices in sepsis, including LDAR (15, 16, 42, 43), RAR (19, 44, 45), UAR (22, 46), and GAR in other critical conditions (47, 48), but none performed head-to-head comparisons across multiple indices with external validation. Our results extend these reports by showing that logLDAR outperforms the other five when tested together. RAR lost significance in the fully adjusted external model (*P* = 0.988), and other indices also lost significance. This suggests that their independent contribution is weaker than that of logLDAR when comprehensive confounders are controlled. Duman et al. (49) suggested that albumin alone might outperform several ratios. Our results partially contrast with this, because logLDAR performed better than albumin alone would predict. This is likely because LDH adds complementary information on tissue injury.

In pairwise AUC comparisons within the derivation cohort, logLDAR was not significantly superior to RAR (*P* = 0.449), UAR (*P* = 0.138) or TAR (P = 0.064). However, it was significantly better than logGAR (*P* < 0.001) and AGAR (*P* = 0.008). At the Youden cut-off (logLDAR = 7.010), the sensitivity and specificity were 51.0% and 69.7% internally. In external validation, these improved to 62.5% and 73.8%, with a negative predictive value of 93.9%. The average cNRI of logLDAR was 0.119 internally and 0.581 externally, far exceeding the other indices. Thus, although logLDAR’s AUC advantage over RAR, UAR and TAR in the derivation cohort was modest, its consistency across cohorts, adjustment models and reclassification metrics makes it the most reliable single prognostic index. We therefore use the term “more consistent prognostic performance” rather than “outperforms” to reflect the limited statistical differences.

From a clinical perspective, the threshold logLDAR > 7 (corresponding to LDAR > 128) provides a simple, bedside-calculable cut-off. Uroseptic patients with logLDAR above this value on ICU admission have a sharply increased 28-day mortality risk. They may warrant intensified organ support, closer hemodynamic monitoring and prompt source control. A value below 7 has a high negative predictive value (93.9% externally), which helps identify lower-risk patients who may avoid overtreatment. For the other indices, higher levels suggest specific organ dysfunction: RAR indicates chronic inflammation/anaemia; AGAR suggests acidosis/electrolyte disturbance; UAR reflects renal dysfunction/catabolism; TAR signals liver impairment; and GAR indicates hyperglycaemia/metabolic stress. However, because these indices lost independent predictive value in external validation, they are better used as adjunctive indicators of organ dysfunction rather than as standalone mortality predictors. It is important to emphasize that logLDAR is a prognostic marker for risk stratification. It should not be interpreted as a direct target for therapy. Any suggestions about “correcting hypoalbuminaemia” or “improving tissue perfusion” remain hypotheses that require prospective interventional studies.

To explore whether combining logLDAR with other features could improve performance, we built an MLP model using eight LASSO-selected variables (including logLDAR, coefficient 0.033, second only to RAR). This model achieved an external AUC of 0.762 (95% CI: 0.687–0.840). This was higher than several individual severity scores, but this comparison is not like-for-like (multivariable model versus single score). SHAP analysis ranked logLDAR as a positive contributor. Calibration and decision curves showed reasonable performance and net benefit. However, a simpler logistic model that combined SOFA with logLDAR achieved a comparable external AUC of 0.782 (DeLong *P* = 0.512 versus the MLP). This suggests that the full neural network offers limited incremental value at this sample size. LASSO selection stability was modest (logLDAR was selected in 46% of bootstrap resamples), which is consistent with the known instability of penalized selection in small samples. The internal validation AUC of the MLP (0.686) was lower than the external AUC (0.762). This counter-intuitive gap may partly reflect differences in case-mix between the two cohorts.

This study has several limitations that require careful interpretation. First, the external validation cohort was relatively small (*n* = 353, 40 events). Both cohorts came from single countries (USA and China), and the Chinese cohort may have distinct clinical characteristics (50, 51). Broader multi-center prospective validation is needed. Second, the retrospective design cannot exclude residual confounding from unmeasured variables, such as nutritional regimens and albumin dose/timing. Third, all indices were based on the first measurement after ICU admission. We did not include dynamic trajectories. Septic patients change rapidly, and serial monitoring might provide richer information. Fourth, the MLP is a “black box” model, which limits interpretability. SHAP partially addresses this, but prospective workflow studies are still required. Fifth, we lacked data on nutritional support, cytokine levels and long-term functional outcomes. Sixth, the optimal cut-off for logLDAR (7.010) was derived from the Youden index in the derivation cohort. Although it was validated externally, the sample size was small. Further calibration across different healthcare settings is needed. Seventh, the definition of urosepsis differed between cohorts. The derivation cohort used ICD codes, while the external cohort used positive urine culture, Sepsis-3 criteria and adjudication by two urologists. The external definition is stricter and the disease spectrum is more concentrated. The higher external AUC (0.735 vs 0.638) may partly reflect this difference in case definition rather than pure cross-center robustness. We could not perform a sensitivity analysis with a harmonized definition because urine culture results were not available in the derivation cohort. Eighth, the fully adjusted Model 3 in the external cohort included about 34 covariates with only 40 events (EPV≈1.2), far below the recommended minimum of 10. This likely led to overfitting and biased the hazard ratio upward (from HR = 1.524 in the parsimonious model to HR = 2.213 in the full model). Therefore, the parsimonious model (EPV = 10) should be considered the primary external validation result. Ninth, the comparison between the 8-feature MLP and individual severity scores is not like-for-like. A multivariable model with SOFA+logLDAR achieved comparable external performance (AUC = 0.782, *P* = 0.512), indicating that the complex model did not provide substantial added value in this dataset.

## Conclusion

This study systematically compared six albumin-derived composite indices for predicting 28-day ICU mortality in urosepsis using the MIMIC-IV database and a real-world external cohort. We provide evidence that LDAR showed more consistent prognostic performance than AGAR, GAR, RAR, TAR and UAR; this consistency remained in external validation, whereas the other five indices lost independent predictive ability. Fine-Gray competing-risks analysis confirmed these findings. logLDAR exhibited a non-linear dose-response relationship with mortality, with risk accelerating sharply after logLDAR > 7, indicating a clear clinical threshold. A multilayer perceptron model built on eight LASSO-selected features (with logLDAR contributing the most) achieved an external AUC of 0.762 (95% CI: 0.687–0.840), showing comparable performance to a multivariable model combining SOFA with logLDAR (AUC = 0.782, DeLong *P* = 0.512). The pathophysiological advantage of logLDAR lies in simultaneously capturing tissue hypoxia/cell injury and nutrition-inflammation depletion. As a simple, readily available bedside biomarker, logLDAR enables early risk stratification in patients with urosepsis. Whether nutritional interventions targeting the pathways captured by logLDAR can improve outcomes requires prospective interventional study. Prospective multi-center studies with harmonized case definitions are needed to further validate its clinical translation value.

## Data Availability

The original contributions presented in this study are included in this article/Supplementary material, further inquiries can be directed to the corresponding authors.

## Supplementary material

Supplementary Figure 1 Kaplan-meier survival curves by albumin-derived indices (external validation cohort). Kaplan-Meier survival curves stratified by derivation cohort median values of six albumin-derived indices in the external validation cohort (*n* = 353). **(A)** log(LDAR), *P* < 0.001; **(B)** log(GAR), *P* = 0.788; **(C)** RAR, P = 0.114; **(D)** AGAR, *P* < 0.001; **(E)** UAR, *P* < 0.001; **(F)** TAR, *P* = 0.041. Red lines = above-median group; Blue lines = below-median group. Shaded areas represent 95% confidence intervals.

Supplementary Figure 2 Restricted Cubic Spline Analysis of Albumin-Derived Indices and ICU Mortality (External Validation Cohort). Restricted cubic spline (RCS) curves in the external validation cohort (*n* = 353). **(A)** log(LDAR), *P* overall < 0.001, P nonlinear = 0.037; **(B)** log(GAR), not significant; **(C)** RAR, not significant; **(D)** AGAR, *P* overall < 0.001, *P* nonlinear = 0.036; **(E)** UAR, *P* overall < 0.001, *P* nonlinear = 0.012; **(F)** TAR, not significant. Knots placed at the 5th, 35th, 65th, and 95th percentiles. Shaded areas represent 95% confidence intervals.

Supplementary Figure 3 Cumulative Incidence Functions for ICU Death and Discharge by logLDAR Group. Cumulative incidence functions (CIF) estimated under the competing-risks framework, stratified by the derivation cohort median logLDAR value. **(A)** Derivation cohort - cumulative incidence of ICU death. **(B)** Derivation cohort - cumulative incidence of ICU discharge alive. **(C)** External validation cohort - cumulative incidence of ICU death. **(D)** External validation cohort - cumulative incidence of ICU discharge alive. Red lines = high logLDAR group; Blue lines = low logLDAR group. Gray’s test *P* < 0.001 for equality of death CIFs in both cohorts.

Supplementary Figure 4 External Calibration Curve for the 8-Feature Prediction Model. Calibration curve in the external validation cohort (*n* = 353). Dashed line = ideal calibration; red line = loess smooth; blue circles = decile groups. Hosmer-Lemeshow goodness-of-fit *P* = 0.124.
