# Supplementary material for: LDAR shows more consistent prognostic performance than other albumin-derived indices for 28-day ICU mortality in critically ill patients with urosepsis: evidence from a dual-cohort retrospective study
Source: Front Med (Lausanne). 2026 Oct 1;13:1915095. doi: 10.3389/fmed.2026.1915095 (PMC13669183; doi:10.3389/fmed.2026.1915095)
Supplement: Supplementary file 1 [file Supplementary_file_1.docx]

**Figure S1: Kaplan-Meier Survival Curves by Albumin-Derived Indices (External Validation Cohort)**

Kaplan-Meier survival curves stratified by derivation cohort median values of six albumin-derived indices in the external validation cohort (n=353). A: log(LDAR), P<0.001; B: log(GAR), P=0.788; C: RAR, P=0.114; D: AGAR, P<0.001; E: UAR, P<0.001; F: TAR, P=0.041. Red lines = above-median group; Blue lines = below-median group. Shaded areas represent 95% confidence intervals.

**Figure S2: Restricted Cubic Spline Analysis of Albumin-Derived Indices and ICU Mortality (External Validation Cohort)**

Restricted cubic spline (RCS) curves in the external validation cohort (n=353). A: log(LDAR), P overall<0.001, P nonlinear=0.037; B: log(GAR), not significant; C: RAR, not significant; D: AGAR, P overall<0.001, P nonlinear=0.036; E: UAR, P overall<0.001, P nonlinear=0.012; F: TAR, not significant. Knots placed at the 5th, 35th, 65th, and 95th percentiles. Shaded areas represent 95% confidence intervals.

**Figure S3: Cumulative Incidence Functions for ICU Death and Discharge by logLDAR Group.** Cumulative incidence functions (CIF) estimated under the competing-risks framework, stratified by the derivation cohort median logLDAR value. A: Derivation cohort - cumulative incidence of ICU death. B: Derivation cohort - cumulative incidence of ICU discharge alive. C: External validation cohort - cumulative incidence of ICU death. D: External validation cohort - cumulative incidence of ICU discharge alive. Red lines = high logLDAR group; Blue lines = low logLDAR group. Gray's test P<0.001 for equality of death CIFs in both cohorts.

**Figure S4: External Calibration Curve for the 8-Feature Prediction Model. Calibration curve in the external validation cohort (n=353).** Dashed line = ideal calibration; red line = loess smooth; blue circles = decile groups. Hosmer-Lemeshow goodness-of-fit P=0.124.
